# Prevalence of schistosomiasis and its association with anemia among pregnant women: a systematic review and meta-analysis

**DOI:** 10.1186/s13071-021-04642-4

**Published:** 2021-03-02

**Authors:** Ishag Adam, Nadiah A. ALhabardi, Osama Al-Wutayd, Ammar H. Khamis

**Affiliations:** 1 Department of Obstetrics and Gynecology, Unaizah College of Medicine and Medical Sciences, Qassim University, Unaizah, Kingdom of Saudi Arabia; 2 Department of Family and Community Medicine, Unaizah College of Medicine and Medical Sciences, Qassim University, Unaizah, Kingdom of Saudi Arabia; 3 Hamdan Bin Mohammed College of Dental Medicine, Mohammed Bin Rashid University of Medicine and Health Sciences, Dubai, United Arab Emirates

**Keywords:** Prevalence, Pregnancy, *Schistosoma haematobium*, *Schistosoma mansoni*

## Abstract

**Background:**

Schistosomiasis is a highly prevalent parasitic disease that can lead to adverse maternal and perinatal outcomes. To our knowledge, there has been no systematic review and meta-analysis of schistosomiasis during pregnancy.

**Methods:**

We followed the Preferred Reporting Items for Systematic Reviews and Meta-Analyses guidelines. Relevant published studies were searched in international databases (PubMed, Science Direct, Scopus, Web of Science, and Google Scholar), from their inception until May 31, 2020. The retrieved studies were assessed for quality using the Modified Newcastle-Ottawa Scale. OpenMeta Analyst software was used for the statistical analysis.

**Results:**

Thirty-two studies enrolling 21024 pregnant women were included in this meta-analysis. All 32 of these studies were conducted in Africa. Of these studies, 19, 11, and 2 investigated *S. mansoni*, *S. haematobium*, and combined *S. mansoni* and *S. haematobium* infections, respectively. The pooled prevalence estimate of schistosomiasis during pregnancy was 13.2% (95 CI 11.0–15.4). A random model was used because of high heterogeneity (Q = 99.14; *P* < 0.001). In subgroup analyses, the pooled prevalence estimate of *S. haematobium* was significantly higher than the pooled prevalence estimates of *S. mansoni* [22.5% (95% CI 1.6–43.5) *vs* 8.7% (95% CI 6.0–11.3, *P* = 0.016), respectively]. The results of meta-regression analyses showed a non-significant difference in the prevalence of schistosomiasis during pregnancy according to the study sample sizes and year of publication. Only six studies evaluated the association between schistosomiasis during pregnancy and anemia. Schistosomiasis was associated with anemia in these six studies (OR = 3.02, 95% = 1.25‒7.28, *P* = 0.014).

**Conclusion:**

The present meta-analysis suggests that schistosomiasis during pregnancy is an existing health problem. This meta-analysis also highlights the lack of data on the determinants and outcomes of schistosomiasis during pregnancy. Preventive measures are needed and could be part of antenatal care in areas endemic with schistosomiasis.
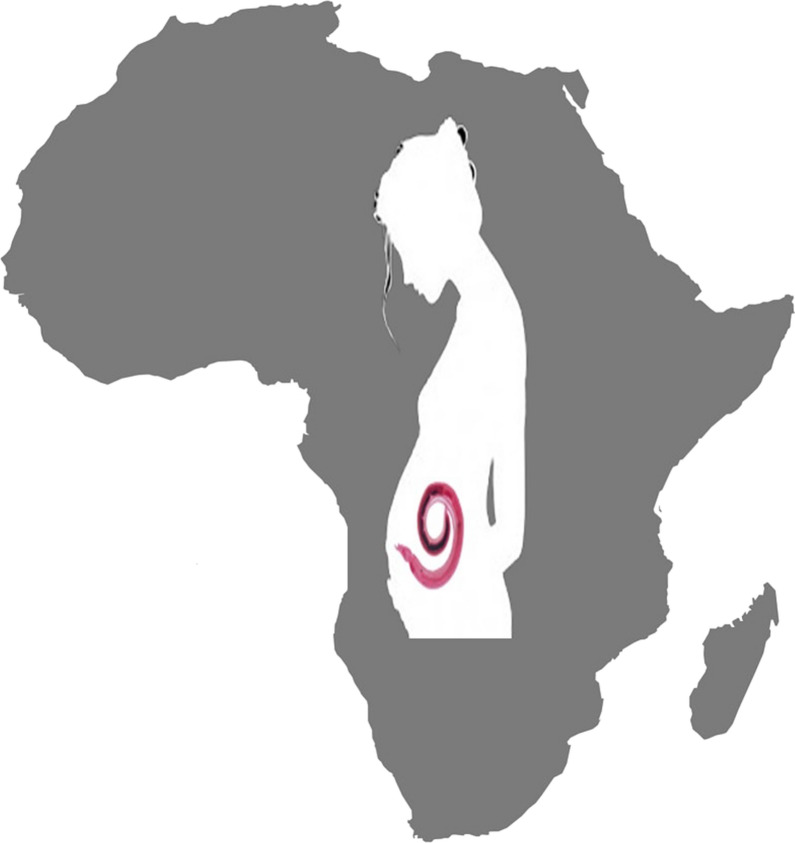

## Introduction

Schistosomiasis, also known as bilharzia, is a waterborne helminthic infection caused by parasitic flatworms belonging to *Schistosoma* spp. blood flukes. It is a highly prevalent parasitic disease worldwide that causes disease in over 200 million people, 90% of whom live in Africa [1]. The life cycle of schistosomes includes freshwater snails as intermediate hosts, from which infective cercariae are released into fresh water and can penetrate unbroken human skin. Schistosomiasis is especially prevalent in countries with tropical climates and limited access to clean water [1]. It was estimated that, in 2014, 40 million women of reproductive age had schistosomiasis (*Schistosoma haematobium, S. japonicum*, and/or *S. mansoni*) [2]*.* During pregnancy, helminth infections, including schistosomiasis, can lead to modulation of the immune response (change from Th1 toward Th2), increasing the susceptibility of pregnant women to various infections, such as influenza, malaria, measles, and toxoplasmosis, and their severity and can further lead to immune modulation in newborns [3, 4]. Previous studies have reported that schistosomiasis in pregnancy is associated with anemia [5–7]. Moreover, schistosomiasis, especially the urogenital type, might lead to adverse outcomes, such as low birth weight deliveries, preterm deliveries, and infertility [8, 9]. Although several studies on schistosomiasis during pregnancy have been conducted in different countries [6–8, 10–38], empirical estimates of the global disease burden during pregnancy are lacking. However, the estimation of the global burden of schistosomiasis during pregnancy is of paramount importance, as it will guide preventive measures and other interventions. The current systematic review and meta-analysis was conducted to estimate the pooled prevalence of schistosomiasis and its association with anemia among pregnant women.

## Methods

We followed the Preferred Reporting Items for Systematic Reviews and Meta-Analyses (PRISMA) guidelines in this study [39]. The included studies were assessed by using the Joanna Briggs Institute Meta-Analysis of Statistics Assessment and Review Instrument (JBI-MAStARI) [40]. Two investigators (NAA and HZH) independently searched and identified eligible studies. If there was disagreement, it was resolved by discussion with a third researcher (IA). The extracted data (author’s name, year of publication, study site, type of study, number of pregnant women with schistosomiasis, and total number of pregnant women enrolled) were recorded in an Excel sheet (Additional file 1).

### Search strategy

A comprehensive literature search was conducted in international databases (PubMed, Science Direct, Scopus, Web of Science, and Google Scholar, from their inception until May 31, 2020) to evaluate the prevalence of schistosomiasis in pregnant women. The searches were performed using MeSH terms either alone or in combination: [“*schistosoma*,” “schistosomiasis,” “bilharzia,” “bilharziasis” OR “*Schistosoma mansoni*” OR “*Schistosoma haematobium*” OR “*Schistosoma japonicum”* AND (“Prevalence” OR “Epidemiology”) AND (“Pregnancy” OR “Pregnant women”) AND (“Anemia” OR “Hemoglobin”)]. The references of the included articles were searched further for more suitable articles.

**Fig. 1:**
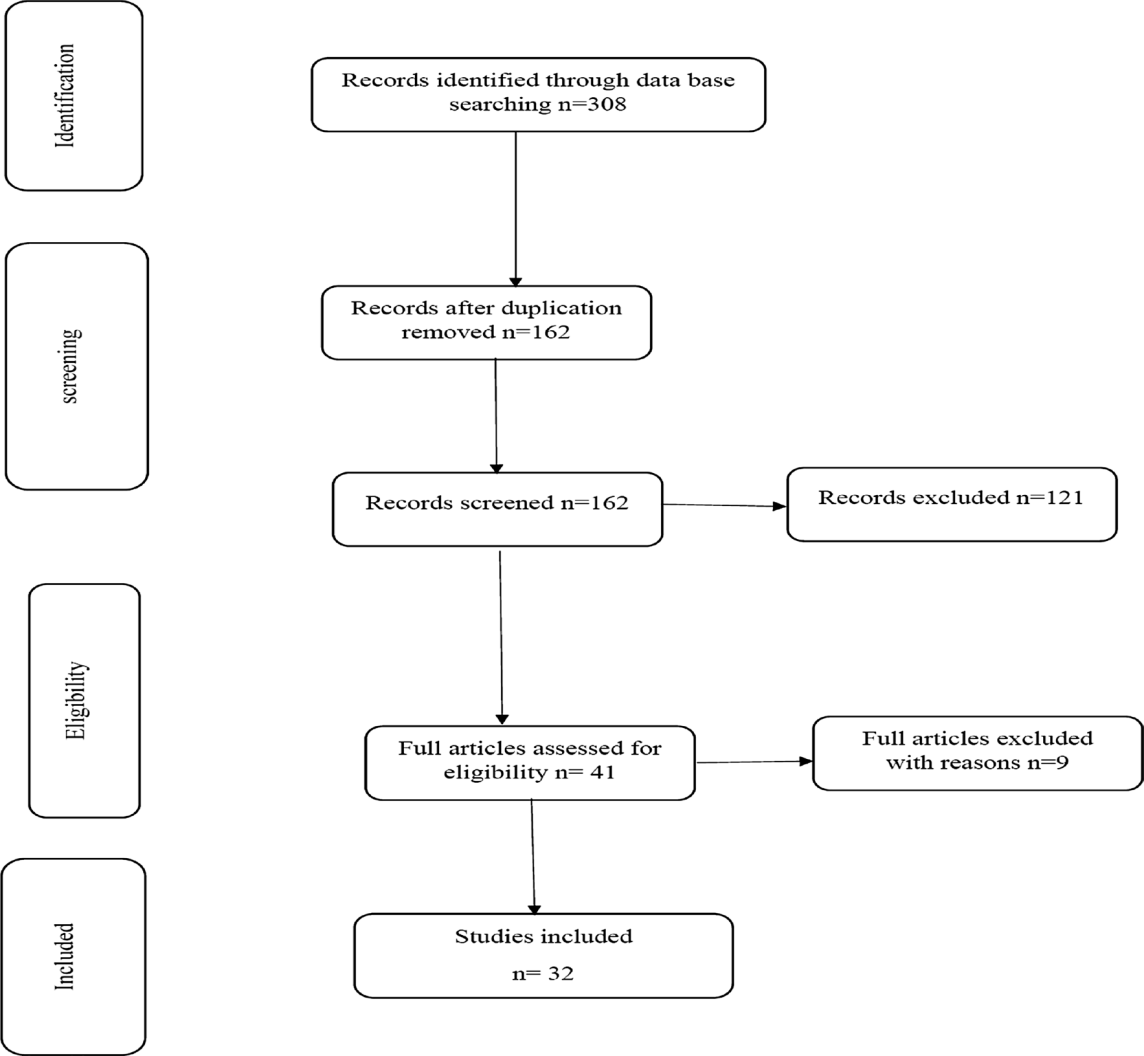
Flow diagram showing the number of articles identified in the systematic review and meta-analysis of the prevalence of schistosomiasis during pregnancy.

Articles were included in the meta-analysis if they met the following inclusion criteria: all cross-sectional studies published in peer-reviewed journals and reporting the prevalence of schistosomiasis in pregnant women, full text or abstract, in the English language, and investigating at least one type/species of schistosomiasis using standard parasitological methods. Case reports, letters, animal studies, duplicates, reviews, and articles in languages other than English were excluded (Fig. 1).

The quality of each study was assessed using the Modified Newcastle–Ottawa Scale (NOS) for cross-sectional or case-control studies [41]. Three major domains—the selection of participants, comparability of study groups, and ascertainment of outcomes of interest in each study—were evaluated by the NOS, with a maximum of nine stars. High-quality studies were assigned if the NOS score was ≥ 7 stars (Table 1).

**Table 1:** Ottawa rating for included studies: (* OR ** means criteria fulfilled/maximum score = 9)

Study	Selection	Comparability	Outcomes	Total
Adewale et al.	****	**	**	8
Ahenkorah et al.	****	**	***	9
Ajanga et al.	****	**	***	9
Alem et al.	***	**	***	8
Anchang-Kimbi et al.	****	**	**	8
Ayoya et al.	****	**	**	8
Bolka et al.	****	**	**	8
Coulibaly et al.	****	**	***	9
Derso et al.	****	**	***	9
Egwunyenga et al.	****	**	**	8
Eyo et al.	****	**	**	8
Fairley et al.	****	**	***	9
Feleke et al.	****	**	**	8
Fuseini et al.	****	**	**	8
Gadoth et al.	****	**	***	9
Gedefaw et al.	****	**	**	8
Kawai et al.	***	**	***	8
Kefiyalew et al.	***	**	**	7
Khalid et al.	***	**	***	8
Kihara et al.	****	**	***	9
Mombo-Ngoma et al.	****	**	**	8
Muhangi et al.	****	**	***	9
Murenjekwa et al.	****	**	**	8
Ndyomugyenyi et al.	****	**	**	8
Ouédraogo et al.	****	**	***	9
Oyeyemi et al.	****	**	***	9
Salawu et al.	****	**	**	8
Siegrist et al.	****	**	**	8
Tay et al.	****	**	***	9
Thigpen et al.	****	**	**	8
Tonga et al.	***	**	***	8
Wepnje et al.	****	**	**	8

### Statistical analysis

We used OpenMeta Analyst software for Windows [42, 43] to perform the meta-analyses. The heterogeneity of the included studies was evaluated according to Cochrane Q and *I*^2^. *I*^2^ values of 25%, 50%, and 75% were considered to represent low, moderate, and high heterogeneity, respectively [44]. The random-effects model was used in case of significant heterogeneity between studies; otherwise, the fixed-effects model (Mantel-Haenszel method) was used for analysis. The pooled prevalence of schistosomiasis and the 95% confidence interval (95% CI) were recorded. Subgroup/meta-regression analysis (size of study, year of publication, and type of infection) was conducted to detect the source of heterogeneity. Moreover, the pooled OR and 95% CI were computed for the association between schistosomiasis and anemia. Funnel plots and Egger’s test were used to assess the publications. A *P*-value was considered statistically significant if it was < 0.05.

## Result

A total of 308 records were identified and 146 duplicated articles were removed. The remaining 162 records were screened using the titles and abstracts, and 121 were excluded. Full texts of 41 articles were then evaluated according to eligibility criteria. Nine articles were also excluded. Finally, 32 articles were included in the study as they passed the eligibility criteria and quality assessment (Fig. 1).

Thirty-two studies enrolling 21,024 pregnant women were included in this meta-analysis [6–8, 10–38]. The sample size ranged from 120 [30] to 4437 pregnant women [19]. All of these studies were of high quality (Table 1).

### Countries

**Fig. 2:**
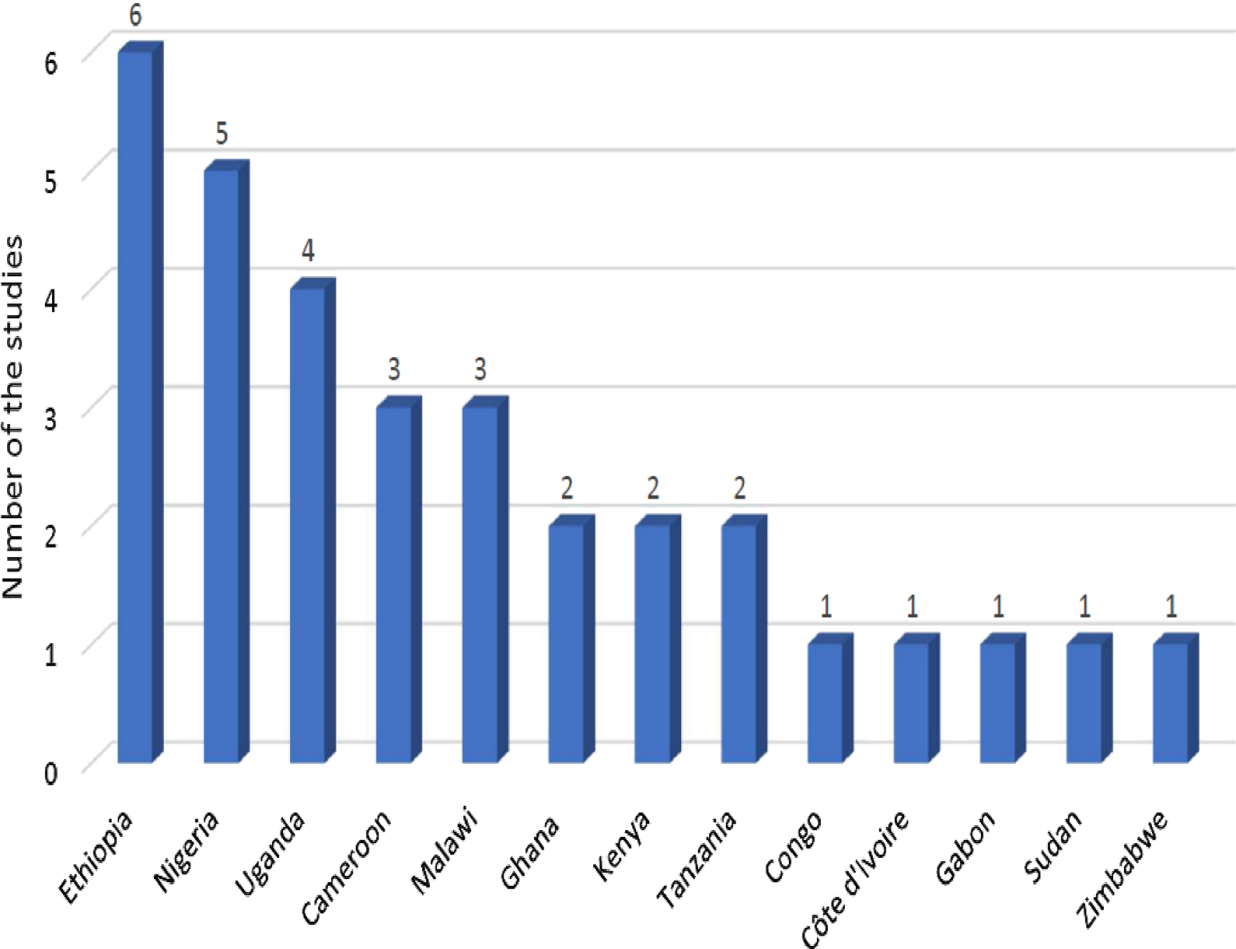
Number of studies included from each country.

All 32 of the studies were conducted in Africa. Six, five, and four studies were conducted in Ethiopia [11, 17, 24, 29, 31, 34], Nigeria [14, 20, 25, 30, 37], and Uganda [6, 12, 16, 27], respectively. One study was conducted in Democratic Republic of Congo [15], Côte d'Ivoire [36], Sudan [10], Gabon [8], and Zimbabwe [19] (Fig. 2).

### Species

Among these 32 studies, 19, 11, and 2 studies investigated *S. mansoni* [7, 10–12, 16, 17, 22, 24, 26–31, 33, 34, 36–38], *S. haematobium* [8, 13–15, 18–21, 25, 32, 35], and combined infections of *S. mansoni* and *S. haematobium* [6, 23], respectively.

### Prevalence

**Fig. 3:**
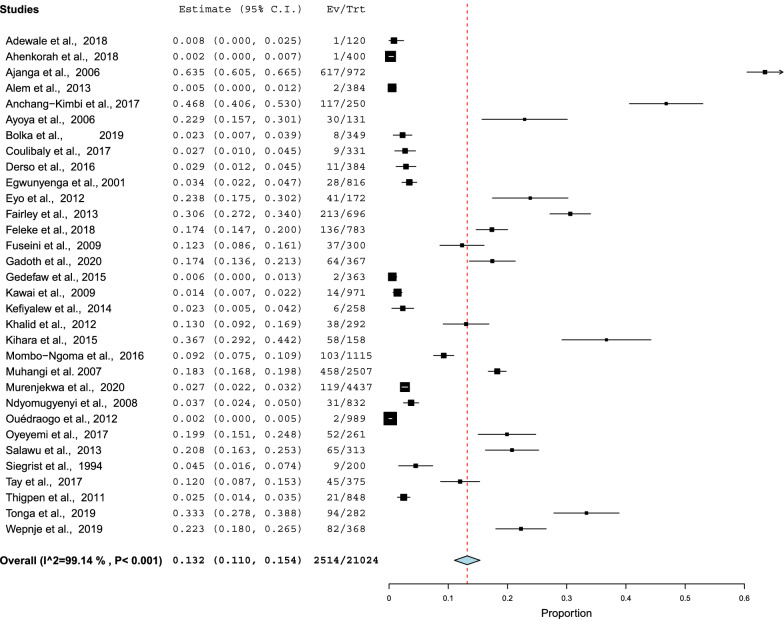
Forest plots of ORs with 95% CIs for the meta-analysis of the pooled prevalence of schistosomiasis using a fixed model.

The prevalence of schistosomiasis ranged from 0% in Ghana [33] to 63.0% in Tanzania [7]. The pooled prevalence estimate of schistosomiasis during pregnancy was 13.2% (95% CI 11.0–15.4). A random model was used because of high heterogeneity (*Q* = 99.14, *P* < 0.001; Fig. 3).

**Fig. 4:**
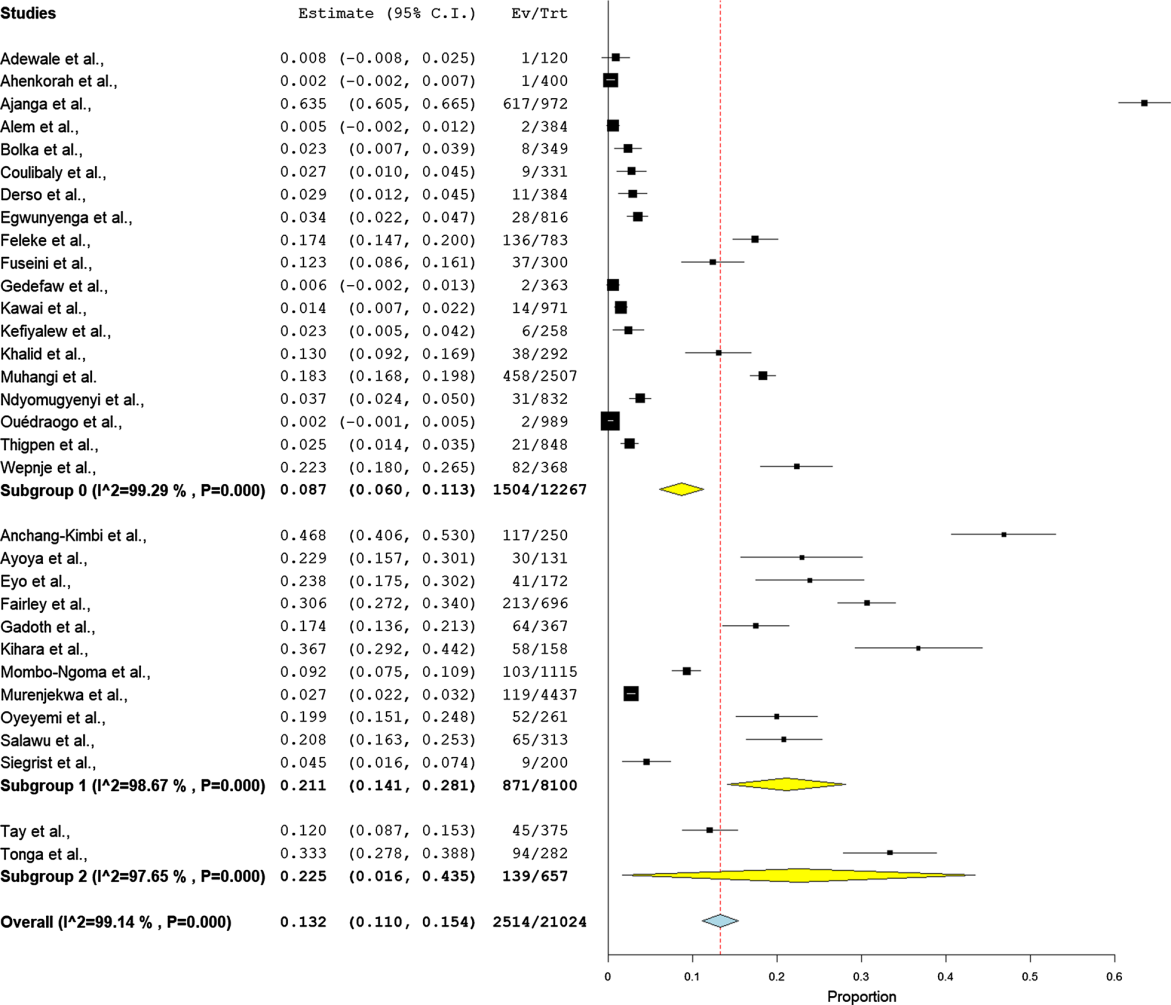
Forest plots of ORs with 95% CIs for the subgroup analysis of the type of infection in a fixed model in the meta-analysis.

In subgroup analyses, the pooled prevalence estimate of *S. haematobium* was significantly higher than the pooled prevalence estimate of *S. mansoni* (22.5% (95% CI 1.6–43.5) *vs* 8.7% (95% CI 6.0–11.3, *P* = 0.016), respectively). A random model was used because of high heterogeneity (*Q* = 99.4, *P* < 0.001; Fig. 4).

**Fig. 5:**
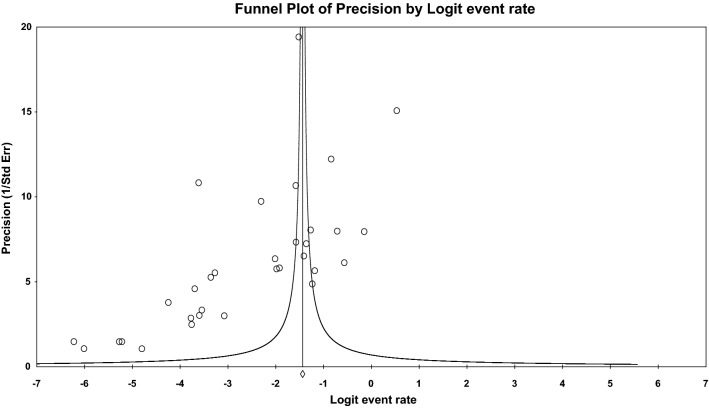
Funnel plot of the publication bias

The results of the meta-regression analyses showed a non-significant difference in the prevalence of schistosomiasis during pregnancy based on the study sample size (coefficient = − 0.001, 95% CI: − 0.001 to 0.001, *P* = 0.538) and year of publication (*C* = − 0.002; 95% CI: − 0.010 to 0.007, *P* = 0.697) (Table 2). Egger’s test showed probable publication bias (*P* = 0.005; Fig. 5).

**Table 2:** Subgroups and metaregression analysis of the factors associated with schistosomiasis during pregnancy

Covariate	Coefficient	95% confidence interval	Standard error	*P*
Size	− 0.001	− 0.001 to 0.001	< 0.001	0.538
Year of publication	− 0.002	− 0.010 to 0.007	− 0.004	0.697
Type of infection	0.097	0.018 to 0.176	0.04	0.016

### Schistosomiasis and anemia

**Fig. 6:**
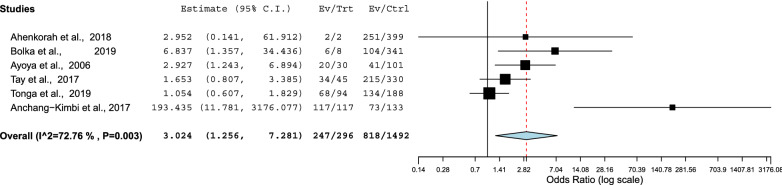
Forest plots of ORs with 95% CIs for the meta-analysis of the association of schistosomiasis with anemia using a fixed model

Only six studies evaluated the association between schistosomiasis during pregnancy and anemia [6, 23, 31–33, 35]. Schistosomiasis was associated with anemia in these six studies (OR = 3.02, 95% = 1.25‒7.28, *P* = 0.014). A fixed model was used (*Q* = 72.7, *P* = 0.003; Fig. 6).

## Discussion

The main finding of the current meta-analysis was that all the retrieved studies on schistosomiasis during pregnancy were conducted in Africa. Moreover, all these studies documented *S. haematobium* and *S. mansoni* infections without any reports of S. *japonicum.* Sub-Saharan African countries account for more than two-thirds (70%) of schistosomiasis cases. *S. haematobium* and *S. mansoni* infections predominate in Africa [45]. It is worth mentioning that epidemiological data on schistosomiasis during pregnancy are generally scarce in sub-Saharan Africa, as the majority of studies are conducted on schoolchildren [46]. We did not identify any articles on the prevalence of schistosomiasis during pregnancy outside of Africa (Southeast Asia of Latin America). Perhaps there are publications on schistosomiasis during pregnancy in these settings, but they are in languages other than English. We included only published articles in English, which could explain the presence of publication bias in our findings. In Latin America and Southeast Asia, the identified reports addressed the pathophysiology rather than the epidemiology of schistosomiasis during pregnancy [4, 47]. However, in these settings in which there are no published articles on schistosomiasis during pregnancy, there are meta-analyses on schistosomiasis available for other age groups [48, 49]. Moreover, a previous study on *S. japonicum* during pregnancy assessed the pathophysiology rather than the prevalence of schistosomiasis during pregnancy [50].

The current meta-analysis showed a pooled prevalence estimate of schistosomiasis during pregnancy of 13.2%. A previous meta-analysis showed a reported pooled prevalence estimate for *S. haematobium* among adults of 54% [51].

Although only six studies assessed the association between schistosomiasis and anemia, the current meta-analysis showed that pregnant women with schistosomiasis were at three times higher risk of anemia (pooled OR = 3.02). Previous studies have reported that schistosomiasis in pregnancy is associated with anemia [5–7]. In a systematic review and meta-analysis, Kassebaum et al. reported that malaria and schistosomiasis were the main conditions that increased the prevalence of anemia [53]. Although the exact pathophysiological mechanism of anemia and its associations with schistosomiasis have yet to be fully explained, hemolysis, inflammatory processes, and bone marrow suppression are plausible explanations for anemia and schistosomiasis [52]. Interestingly, few studies (six) assessed the association between schistosomiasis during pregnancy and anemia, and there was no available meta-analysis of this topic. It is possible that researchers do not feel it is important to document this association, and it might be considered an established fact rather than an area for research. A recent meta-analysis showed that mass deworming during pregnancy reduces maternal anemia by 23%; however, there is no evidence of it having an impact on any other maternal or pregnancy outcomes [54].

Other effects of schistosomiasis on pregnancy outcomes, such as reduced maternal-to-infant antibody transfer [50, 55], infertility, preterm deliveries, and lower birth weights [9], need to be addressed. The adverse effects of schistosomiasis during pregnancy could be explained by the modulation of the immune response (change from Th1 toward Th2), which could be accompanied by immune modulation in newborns [3, 4].

Researchers might have investigated schistosomiasis epidemiology and its treatment (praziquantel) among children [56]. Regarding praziquantel safety during pregnancy, it was long indicated that “no data during pregnancy” were available. Following our reports on praziquantel safety during pregnancy[57, 58], it has come to be indicated as a safe, effective drug [59].

### Limitations of the study

Many potential risk factors, such as age and parity, were not assessed in these studies. Furthermore, the cross-sectional nature of the included studies is not amenable to the dissection of the causal relationships between schistosomiasis and anemia.

## Conclusion

The present meta-analysis suggests that schistosomiasis during pregnancy is an existing health problem. This meta-analysis also highlights the lack of data on the determinants and outcomes of schistosomiasis during pregnancy. Preventive measures are needed and could be part of antenatal care in areas endemic for schistosomiasis.

## Supplementary Information

**Additional file 1.** Characteristics of all studies included in this systematic review and meta-analysis of the prevalence of schistosomiasis during pregnancy.

## Data Availability

All data are included in this paper**.**

## Abbreviations

AOR: Adjusted odds ratio
CI: Confidence interval
GDM:SD: Deviation
